# Development of an Assay Involving Loop-Mediated Isothermal Amplification with a Lateral Flow Dipstick for Detection of the *vanA* Gene in Vancomycin-Resistant Enterococci  

**DOI:** 10.3390/ijms27156575

**Published:** 2026-07-23

**Authors:** Saranthum Phurijaruyangkun, Pongbun Tangjitrungrot, Sawanya Pongparit, Naiyana Wattanakul, Bajaree Jantrapanukorn, Rungnapa Veeramano, Suphitcha Augkarawaritsawong, Khurawan Kumkrong, Thitichot Sirothot, Pornpun Jaratsing, Supatra Areekit, Kankanit Rattanathanawan, Somchai Santiwatanakul, Kantima Choosang, Kosum Chansiri

**Affiliations:** 1 Faculty of Medical Technology, Rangsit University, Pathum Thani 12000, Thailand; saranthum.p@rsu.ac.th (S.P.); sawanya.p@rsu.ac.th (S.P.); bajaree.j@rsu.ac.th (B.J.); rungnapa.v@rsu.ac.th (R.V.); suphitcha.a@rsu.ac.th (S.A.); khurawan.k@rsu.ac.th (K.K.); thitichot.s@rsu.ac.th (T.S.); 2 Center of Excellence in Biosensors, Panyananthaphikhu Chonprathan Medical Center, Srinakharinwirot University, Nonthaburi 11120, Thailand; pongbun@g.swu.ac.th (P.T.); pornpunj@g.swu.ac.th (P.J.); titi41@yahoo.com (S.S.); 3 Nopparatrajathanee Hospital, Bangkok 10230, Thailand; naiya447@gmail.com; 4 Innovative Learning Center, Srinakharinwirot University, Bangkok 10110, Thailand; supatraa@g.swu.ac.th (S.A.); kankanit@g.swu.ac.th (K.R.)

**Keywords:** vancomycin-resistant enterococci, *vanA* gene, loop-mediated isothermal amplification, lateral flow dipstick

## Abstract

Vancomycin-resistant enterococci (VRE) have emerged as major nosocomial pathogens worldwide, primarily due to the acquisition of the *vanA* gene, which confers high-level resistance to vancomycin. Rapid detection of the *vanA* gene, which is associated with vancomycin resistance, is crucial for timely clinical management and infection control. Through this research, we aimed to develop an assay combining loop-mediated isothermal amplification and a lateral flow Dipstick (LAMP-LFD) for the rapid detection of the *vanA* gene in VRE. Genomic DNA was extracted and amplified from 100 clinical urine samples by LAMP targeting *vanA* under optimized, constant-temperature conditions, with the amplified product then detected using a lateral flow Dipstick assay based on probe pairing. The diagnostic performance of the LAMP-LFD method was evaluated and compared with a conventional PCR reference method. The LAMP-LFD assay successfully detected the *vanA* gene in all positive samples, with negative samples showing no assay cross-reactivity with 12 species of bacteria commonly found in clinical settings. The developed LAMP–LFD assay showed promising diagnostic performance for detecting the *vanA* gene in *Enterococcus* isolates, demonstrating complete agreement with the reference PCR method in this study. Owing to its rapid turnaround time, simplicity, and visual readout, the assay has potential as a molecular screening tool for VRE. However, further validation in larger, multicenter studies and comparison with phenotypic antimicrobial susceptibility testing are required before routine clinical implementation.

## 1. Introduction

Vancomycin-resistant *Enterococcus* (VRE) has emerged as a significant cause of nosocomial infections worldwide, particularly among immunocompromised patients and those with prolonged hospital stays. While members of the genus *Enterococcus*, especially *Enterococcus faecalis* and *Enterococcus faecium*, are part of the normal gastrointestinal flora, they have increasingly become opportunistic pathogens responsible for endocarditis and bloodstream and urinary tract infections. The rapid dissemination of antimicrobial resistance in these organisms poses a major public health concern due to the limited therapeutic options and increased rates of morbidity and mortality [1,2].

Vancomycin resistance in *Enterococcus* is mediated by several gene clusters, including *vanA*, *vanB*, and *vanC*, and less common variants such as *vanD*, *vanE*, *vanG*, *vanL*, *vanM*, and *vanN*. Among these, *vanA* and *vanB* are the most clinically relevant and widely distributed worldwide, with *vanA* conferring high-level resistance to both vancomycin and teicoplanin, while *vanB* exhibits variable resistance levels [3]. These resistance determinants are frequently located on mobile genetic elements, facilitating horizontal gene transfer between bacterial populations.

The principal mechanism of vancomycin resistance in enterococci involves the acquisition of resistance gene clusters, with the *vanA* gene being the most clinically significant and widely distributed determinant. The *vanA* operon encodes enzymes that alter the terminal D-Ala-D-Ala structure of peptidoglycan precursors to D-Ala-D-Lac, resulting in a marked reduction in vancomycin binding affinity and, consequently, high-level resistance. Notably, vanA-type resistance is often associated with transferable genetic elements such as transposon Tn1546, facilitating horizontal gene transfer between bacterial populations and contributing to the rapid spread of resistance in healthcare settings [4,5].

Conventional methods for detecting VRE, including culture-based antimicrobial susceptibility testing and phenotypic assays, are time-consuming, sometimes requiring 24–72 h to yield results. Moreover, phenotypic methods may fail to accurately distinguish between different resistance genotypes or to detect low-level resistance, potentially leading to delayed diagnosis and inappropriate antimicrobial therapy. Therefore, rapid and accurate detection of the *vanA* gene is essential for effective infection control, surveillance, and clinical management [6,7].

Molecular diagnostic techniques have revolutionized the detection of antimicrobial resistance genes by offering high sensitivity and specificity and rapid turnaround times. Polymerase chain reaction (PCR)-based assays remain the gold standard for the detection of resistance genes such as *vanA*; however, they require sophisticated equipment, skilled personnel, and controlled laboratory environments, limiting their applicability in resource-limited settings and point-of-care testing (POCT) scenarios [8,9].

To overcome these limitations, isothermal amplification methods, such as loop-mediated isothermal amplification (LAMP), have been developed as promising alternatives. LAMP enables rapid DNA amplification under constant-temperature conditions, eliminating the need for specialized equipment (e.g., a PCR machine). while maintaining high analytical performance. The integration of LAMP with visual detection systems, including colorimetric indicators and lateral flow dipsticks (LFDs), further enhances its potential as a user-friendly and field-deployable diagnostic tool. These advantages make LAMP-based assays particularly suitable for early VRE detection in clinical and low-resource settings [10,11]. However, despite the availability of conventional PCR and culture-based methods, there remains a significant gap in the development of rapid, simple, and equipment-free diagnostic assays specifically targeting the *vanA* gene, which is the most clinically important determinant of high-level vancomycin resistance in enterococci. 

In this study, we aimed to develop and evaluate LAMP-LFD, a molecular diagnostic method for the rapid detection of the *vanA* gene in vancomycin-resistant enterococci. The isolates were routinely identified by conventional biochemical methods and antimicrobial susceptibility testing before being included in the evaluation of the LAMP-LFD assay. The proposed approach focuses on improving sensitivity, specificity, user-friendly visual interpretation, and turnaround time while maintaining simplicity and cost-effectiveness when compared with conventional culture and PCR, thereby facilitating its potential application as a point-of-care diagnostic tool and supporting infection control strategies.

## 2. Results

### 2.1. PCR Assays Verification

**Figure 1:**
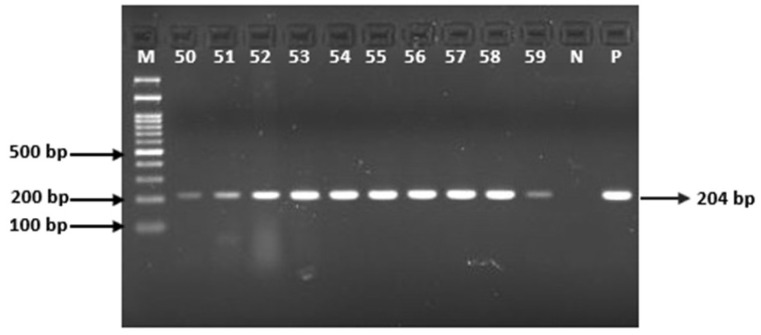
The optimization of annealing temperature conditions from 50 to 59 °C. Lane “M” represents Hyper Ladder 100 bp Plus (Bioline Reagents Ltd., London, UK); “P” and “N” represent positive and negative controls, respectively.

The annealing temperature for PCR amplification was optimized to determine the most suitable condition for specific amplification of the *vanA* target gene. The annealing temperature for PCR amplification was varied from 50 to 59 °C in 1 °C gradations, and the intervals were compared to determine the optimal temperature. The temperature that yielded the most distinct 204 bp PCR amplicon band without the presence of non-specific bands was identified as 57 °C (Figure 1).

### 2.2. Optimization of LAMP Amplification

The LAMP amplification conditions were optimized to determine the best parameters for specific detection of the *vanA* gene. MgSO_4_ concentration, incubation temperature, and reaction time were evaluated to achieve strong amplification with high specificity and minimal non-specific products. The optimal conditions were selected based on the characteristic ladder-like band pattern observed by agarose gel electrophoresis and clear results for subsequent LAMP-LFD detection.

**Figure 2:**
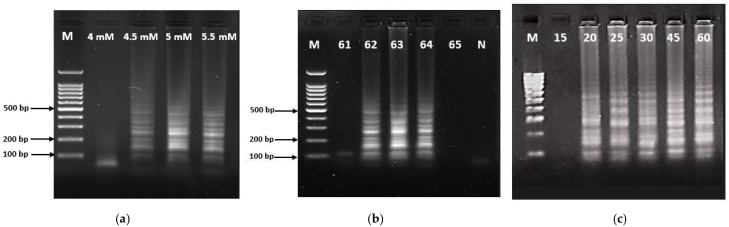
(**a**) LAMP assay optimization of MgSO_4_ in the range of 4–5.5 mM. Lane “M” represents Hyper Ladder 100 bp Plus (Meridian Bioscience, London, UK). (**b**) The optimization of thermal conditions between 61 and 65 °C. Lane “M” represents Hyper Ladder 100 bp Plus (Bioline Reagents Ltd., London, UK), and “N” is the negative control. (**c**) The optimal reaction durations were observed at 20- and 60-min. Lane “M” represents Hyper Ladder 100 bp (Bioline Reagents Ltd., London, UK).

Magnesium sulfate (MgSO_4_), a critical component of the LAMP reaction, was optimized by testing concentrations ranging from 4.0 to 5.5 mM. The best amplification performance was observed at 5.0 mM, which was selected as the optimal concentration (Figure 2a). The optimal amplification temperature for LAMP was determined with a gradient from 61 to 65 °C. The amplification patterns with ladder band clarity visible at 63 °C demonstrate high LAMP assay amplification efficiency. The optimal reaction temperature (63 °C) was selected because it consistently produced the strongest amplification signal, with clear ladder-like bands characteristic of LAMP products, without detectable nonspecific amplification. The result was reproducible in repeated experiments (Figure 2b). LAMP was conducted at 63 °C for 15, 20, 25, 30, 45, and 60 min in order to determine the optimal reaction time. Amplification products were first detectable after 20 min, indicating that the assay was capable of rapid amplification. However, amplification at 20, 25, and 30 min was not consistently observed in all replicate experiments, whereas 45 and 60 min yielded positive amplification in all three independent replicates. Therefore, 45 min was selected as the optimal reaction time because it represented the shortest incubation period that consistently produced reproducible amplification. (Figure 2c).

### 2.3. The Optimal Conditions for LAMP-LFD

To detect LAMP products, the LAMP reaction was combined with a lateral flow dipstick system into the LAMP–lateral flow dipstick (LAMP-LFD) rapid detection method. The dipstick comprised sample, application, and absorbent pads, where the application pad was functionalized with colloidal gold-conjugated rabbit anti-FITC antibodies, and test and control lines containing biotin ligands and immobilized antibodies specific to anti-FITC antibodies, respectively.

**Figure 3:**
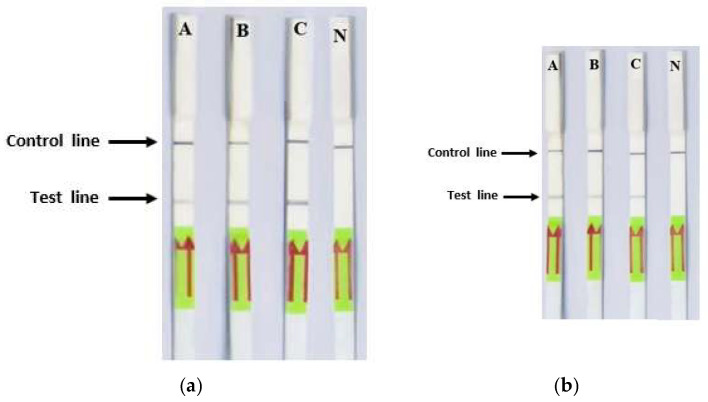
(**a**) Different concentrations of FITC-DNA probe were tested for LAMP-LFD. (A) 5 pmol; (B) 10 pmol; (C) 20 pmol; N—negative control. (**b**) LAMP products from 50 ng of VRE gDNA hybridized with 20 pmol of FITC probe and detected using lateral flow dipsticks via incubation at 63 °C for 5, 10, and 15 min (strips A–C), respectively. Strip N is the negative control.

**Figure 4:**
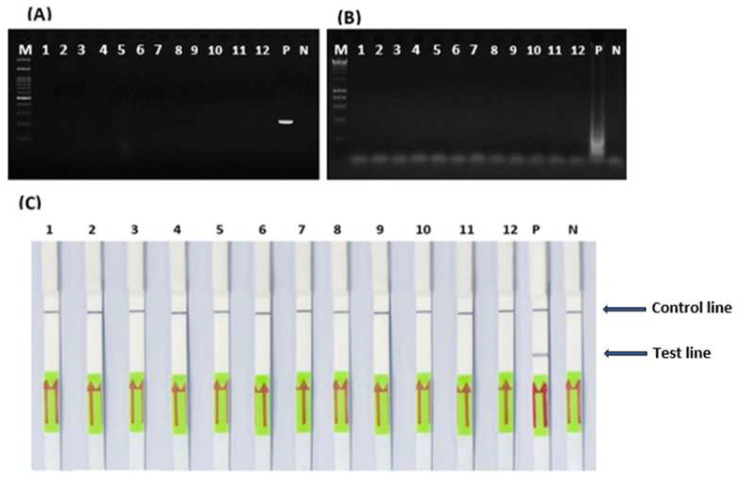
Lane “M” represents DNA ladder marker Hyper Ladder 100 bp Plus (Bioline Reagents Ltd., UK). 1—*E. faecalis* ATCC^®^ 29212 (VSE); 2—vancomycin-resistant *E. faecalis* ATCC^®^ 51299 (harboring *vanB*); 3—oxacillin-resistant *S. aureus* ATCC^®^ 43300 (MRSA); 4—*S. aureus* ATCC^®^ 25923; 5*—S. pneumoniae* ATCC^®^ 49619; 6*—Escherichia coli* ATCC^®^ 25922; 7—Acinetobacter lwoffii ATCC^®^ 15309; 8—*E. cloacae* ATCC^®^ 23355; 9—carbapenem-resistant *K. pneumoniae* ATCC^®^ BAA-1705; 10—*K. aerogenes* ATCC^®^ 13048; 11—*P. aeruginosa* ATCC^®^ 27853; 12—*B. cepacia* ATCC^®^ 25416; “P” is the positive control and “N” is the negative control. (**A**) PCR with agarose gel electrophoresis (PCR-AGE). (**B**) LAMP with agarose gel electrophoresis (LAMP-AGE). (**C**) LAMP combined with lateral flow detection (LAMP-LFD).

Different FITC-DNA probe concentrations were tested for the LAMP-LFD assay. Here, optimization is necessary to avoid the high-dose hook effect, which occurs when an excessive amount of labeled product is introduced into the LFD. In this study, an FITC-probe concentration of 20 pmol was considered optimal, as it provided a clear signal (Figure 3). In order to determine the optimal hybridization time, LAMP products were mixed with 1 µL of 20 pmol of FITC-DNA probe followed by further incubation at 63 °C for 5, 10, or 15 min to allow hybridization to take place. During the optimization of the hybridization parameters, incubation times of 5, 10, and 15 min were evaluated. No observable differences in signal intensity or band clarity were detected across these time points at the lateral flow test line. This indicates that hybridization between the FITC-labeled primer and the target LAMP amplicons reaches thermodynamic and kinetic saturation within 5 min. Consequently, a 5-min hybridization time was selected for the final protocol (Figure 4); therefore, we used LAMP products hybridized with FITC- DNA probe incubated at 63 °C for 5 min for further analysis.

### 2.4. Analytical Specificity Test

PCR, LAMP, and LAMP-LFD assay specificity was determined using *Staphylococcus aureus* ATCC^®^ 25923, oxacillin-resistant *S. aureus* ATCC^®^ 43300 (MRSA), *Enterococcus faecalis* ATCC^®^ 29212 (VSE), vancomycin-resistant *E. faecalis* ATCC^®^ 51299 (harboring *vanB*), *Streptococcus pneumoniae* ATCC^®^ 49619, *Escherichia coli* ATCC^®^ 25922, *Acinetobacter lwoffii* ATCC^®^ 15309, *Enterobacter cloacae* ATCC^®^ 23355, carbapenem-resistant *Klebsiella pneumoniae* ATCC^®^ BAA-1705, *K. aerogenes* ATCC^®^ 13048*, Pseudomonas aeruginosa* ATCC^®^ 27853, and *Burkholderia cepacia* ATCC*^®^ 25416*. All assays showed no cross-reactivity with non-target bacterial species and the *vanB*-harboring strain (Figure 4A–C).

### 2.5. Analytical Sensitivity Test

**Figure 5:**
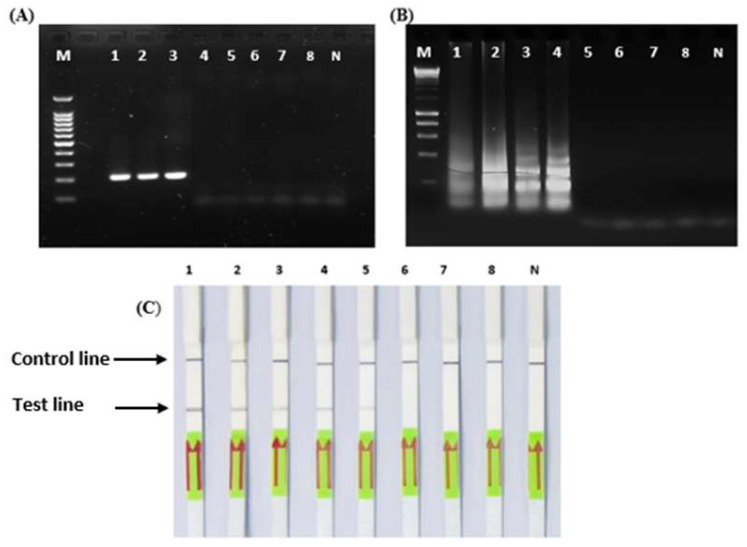
Lane “M” represents the DNA ladder marker Hyper Ladder 100 bp Plus (Bioline Reagents Ltd., UK). DNA 10-fold dilutions: 1: 28.52 ng/µL; 2: 2.852 ng/µL; 3: 285.2 pg/µL; 4: 28.52 pg/µL; 5: 2.852 pg/µL; 6: 285.2 fg/µL; 7: 28.52 fg/µL; 8: 2.852 fg/µL. N: Negative control. (**A**) PCR with agarose gel electrophoresis (PCR-AGE). (**B**) LAMP with agarose gel electrophoresis (LAMP-AGE). (**C**) LAMP combined with lateral flow detection (LAMP-LFD).

The analytical sensitivity of PCR, LAMP, and LAMP-LFD was determined using a 10-fold serial dilution of *E. faecium* ATCC^®^ 700221 from 28.52 ng/µL to 2.852 fg/µL. The PCR, LAMP, and LAMP-LFD assays’ limits of detection were 285.2 pg/uL, 28.52 pg/µL (Lane 4), and 28.52 pg/µL, respectively (Figure 5A–C).

### 2.6. Application of LAMP-LFD to Enterococcus Clinical Isolates

A total of 100 clinical isolates from urine specimens were analyzed and classified as VRE or VSE based on routine antimicrobial susceptibility testing, including vancomycin MIC interpretation according to CLSI 2024 guidelines. These archived isolates were subsequently used for molecular detection of the *vanA* gene and comprised 52 *vanA*-positive and 48 *vanA*-negative *Enterococcus* isolates (see Supplementary Figure S3). As shown in Table 1, the LAMP–LFD assay demonstrated complete concordance with both conventional PCR and LAMP for the detection of *vanA*-positive VRE isolates. The assay achieved a diagnostic sensitivity, specificity, accuracy, positive predictive value (PPV), and negative predictive value (NPV) of 100%, with a total turnaround time of approximately 1–1.5 h, including DNA extraction. Consequently, the positive likelihood ratio (LR+) was infinite (∞), whereas the negative likelihood ratio (LR−) was 0.

A comparison between the conventional LAMP assay and the LAMP–LFD assay showed complete agreement, with both methods yielding identical results for all 100 clinical isolates. The comparative diagnostic performance of the two methods is summarized in Table 2, alongside 95% confidence intervals (CIs) estimated using the exact Clopper–Pearson binomial method. Despite all performance estimates being 100%, the corresponding CIs ranged from 92.6% to 100% for specificity and NPV, and from 93.2% to 100% for sensitivity and PPV. These relatively narrow confidence intervals reflect the robustness of the assay, although the lower bounds indicate the inherent uncertainty associated with the sample size.

## 3. Discussion

*Enterococcus* spp. are Gram-positive cocci that constitute part of the normal gastrointestinal and genitourinary tract microbiota; however, they are also recognized as important opportunistic pathogens responsible for a wide range of nosocomial infections. Among them, *Enterococcus faecalis* and *Enterococcus faecium* are the most clinically significant species, frequently associated with urinary tract infections, bacteremia, and endocarditis. Over the past decades, the emergence and global dissemination of VRE have become a major public health concern due to increasing antimicrobial resistance, limited therapeutic options, and elevated mortality rates [3].

Rapid and accurate VRE detection, particularly of strains harboring *vanA*, is essential for effective infection control and appropriate antimicrobial therapy. Conventional culture-based methods are labor-intensive and time-consuming, whereas polymerase chain reaction (PCR), although highly sensitive and specific, requires specialized equipment and technical expertise. Real-time PCR assays have demonstrated excellent diagnostic performance for *vanA* detection and are increasingly used in clinical settings [12]; however, there remains a growing need for alternative molecular approaches that combine high diagnostic performance with simplicity and rapid turnaround time.

Loop-mediated isothermal amplification (LAMP) has emerged as a promising alternative for rapid nucleic acid detection. Previous studies have demonstrated that LAMP assays targeting the *vanA* gene provide high sensitivity and specificity, often exceeding those of conventional PCR, while requiring minimal equipment [13]. In addition, LAMP enables rapid amplification under isothermal conditions and allows for direct detection from clinical samples.

In the present study, a *vanA*-targeting LAMP assay coupled with a lateral flow dipstick (LAMP–LFD) was developed for the rapid molecular detection of vancomycin-resistant *Enterococcus* (VRE). All clinical isolates were initially characterized by conventional phenotypic vancomycin susceptibility testing before molecular analysis. The LAMP–LFD assay was subsequently evaluated for detecting the *vanA* gene in these phenotypically characterized isolates, using PCR as the molecular reference method. The assay employs Bst DNA polymerase to amplify the target DNA under isothermal conditions, and the amplification products are visualized on a lateral flow dipstick, enabling rapid, equipment-free result interpretation without the need for agarose gel electrophoresis.

The assay’s analytical sensitivity was evaluated using 10-fold serial dilutions of genomic DNA from *E. faecium* ATCC 700221, and the limit of detection was found to be 28.52 pg/µL for both LAMP and LAMP-LFD—10-fold more sensitive than PCR. These findings are consistent with those of Huang, Q.Q., et.al., who developed a LAMP assay for *vanA*-positive *Enterococcus faecium* and similarly reported that LAMP exhibited greater analytical sensitivity than conventional PCR for detecting the *vanA* gene [13]. 

Specificity testing using non-VRE strains showed no cross-reactivity, further confirming the assay’s robustness. In the present study, the LAMP–LFD assay showed no cross-reactivity with non-VRE strains, demonstrating excellent analytical specificity for the *vanA* gene. These findings are consistent with those reported by Kim, H.J., et.al., who developed a LAMP assay targeting the *vanA* gene in *Enterococcus* spp. and similarly observed no amplification among non-*vanA* isolates. [14].

In this study, complete concordance was observed among PCR, LAMP, and LAMP-LFD assays for *vanA* detection, with all 52 VRE isolates testing positive and all 48 VSE isolates testing negative across the three methods. No false-positive or false-negative results were observed, indicating that both LAMP-based methods achieved diagnostic performance comparable to that of PCR. This finding is consistent with other isothermal amplification platforms, such as recombinase polymerase amplification combined with lateral flow detection, which demonstrated 100% sensitivity and specificity for *vanA* detection [15].

The high level of agreement observed in this study may be attributed to optimized reaction conditions and careful primer design targeting conserved regions of the *vanA* gene. Our findings are consistent with a previous report demonstrating that LAMP assays provide robust, reproducible, and highly sensitive detection of antimicrobial resistance genes [13]. Published studies specifically evaluating LAMP–LFD assays for *vanA* detection are currently limited. Most previous investigations have focused on conventional LAMP, PCR-based methods, or commercial molecular assays. Therefore, direct comparisons with identical assay formats remain challenging. Nevertheless, the present study extends the existing evidence by demonstrating that integrating LAMP with probe-based LFD detection preserves the high analytical specificity and diagnostic performance of the LAMP assay while providing a simple visual detection platform suitable for laboratories with limited resources.

Although the present study demonstrated excellent diagnostic performance, with all evaluated parameters reaching 100%, these findings should be interpreted cautiously in light of several inherent limitations. The infinite positive likelihood ratio (LR+) observed in this study resulted from the absence of false-positive results and reflects the complete agreement between the LAMP–LFD assay and the reference PCR method. However, this estimate may be influenced by the relatively limited sample size and should not be interpreted as evidence of perfect diagnostic performance. Furthermore, PCR was used as the molecular reference method rather than phenotypic antimicrobial susceptibility testing, which remains the reference standard for confirming vancomycin resistance. Therefore, further validation using larger, multicenter collections of clinical isolates and comparison with phenotypic susceptibility testing are warranted to establish the clinical performance and generalizability of the LAMP–LFD assay [1,2]. One of the primary concerns is the relatively limited sample size. The study included a total of 100 clinical specimens, comprising 52 positive and 48 negative cases. While this sample size is adequate for preliminary evaluation, it may not fully capture the variability encountered in real-world clinical settings [16,17]. Consequently, the observed perfect agreement may reflect an overestimation of diagnostic performance, particularly in the absence of borderline or challenging cases such as samples reflecting low pathogen load or early-stage infection, or those affected by interfering substances [18,19]. Moreover, the width of the 95% confidence intervals, despite high point estimates, indicates a degree of statistical uncertainty that is typical in studies with constrained sample sizes [20]. For instance, the lower bounds of the sensitivity and specificity confidence intervals did not reach 100%, highlighting the potential variability that may emerge with a larger and more diverse cohort [5,6]. This underscores the importance of cautious interpretation and the need to avoid overgeneralization of the results [16]. Another critical limitation is the lack of external validation. The current evaluation was conducted within a single study setting, which may introduce biases related to sample selection, laboratory conditions, and operator expertise [17,21]. External validation using independent cohorts from different geographic regions, healthcare settings, and patient populations is essential to confirm the assay’s robustness, reproducibility, and generalizability [21,22]. In particular, multicenter studies would provide a more comprehensive assessment of performance across varying epidemiological contexts and operational conditions [22]. Future studies should therefore aim to include larger sample sizes with a broader spectrum of clinical presentations, including asymptomatic individuals, early infections, and cases with potential cross-reactivity [4,8]. Additionally, prospective validation in point-of-care settings would further support the clinical utility and implementation potential of the assay [18,21]. Such efforts are crucial to establish the reliability of the method before its integration into routine diagnostic workflows [16,22].

Although the LAMP-LFD assay demonstrated excellent diagnostic performance, LAMP technology has inherent limitations, including the risk of carryover aerosol contamination, complex primer design, and potential non-specific amplification. These issues were minimized in this study through careful primer optimization and optimized reaction conditions; however, strict contamination control measures, including closed-tube workflows and physical separation of pre- and post-amplification processes, remain essential. Future studies should further evaluate the assay in multicenter clinical settings and incorporate contamination-control strategies, such as UDG/dUTP systems, to enhance robustness and reliability.

## 4. Materials and Methods

### 4.1. Specimen Collection and Bacterial Strains

This study utilized a dual-consecutive sampling method to conduct laboratory-based surveillance of *Enterococcus* species. All isolates were retrieved from clinical urine specimens processed by the microbiology laboratory at Nopparat Rajathanee Hospital (Bangkok, Thailand). Inclusion and Exclusion Criteria: To eliminate duplicate data and prevent statistical skewing, only the first isolate per unique patient was included in the study. Isolates were categorized into two distinct, complete cohorts based on antimicrobial susceptibility testing:

A total of 100 *Enterococcus* isolates were collected from clinical urine specimens between March 2025 and April 2026 at Nopparat Rajathanee Hospital, Bangkok, Thailand, comprising 52 vancomycin-resistant *Enterococcus* (VRE) and 48 vancomycin-susceptible *Enterococcus* (VSE) strains. In this study, *Enterococcus faecium* (ATCC 700221) harboring the *vanA* gene, was used as a positive control. To evaluate the specificity of the PCR, LAMP, and LAMP–LFD assays for *vanA* detection, a panel of bacterial species was included, consisting of five Gram-positive and seven Gram-negative organisms commonly associated with urinary tract infections. Additionally, one reference strain, *Enterococcus faecalis* (ATCC 51299) carrying the *vanB* gene, was included. Ethical approval for this study was obtained from the Rangsit University Institutional Review Board (RSU-ERB2025/135.2705). The study was conducted using anonymized residual urine specimens collected as part of routine clinical care. The requirement for informed consent was waived by the Institutional Review Board because no identifiable patient information was collected or used. 

### 4.2. Genomic DNA Preparation

Genomic DNA was extracted from all culture strains using Presto Mini gDNA Bacterial Extraction Kits (Geneaid Biotech Ltd., New Taipei City, Taiwan, China) according to the manufacturer’s Quick Protocol [23]. The DNA purity and concentration were measured with a NanoDrop 2000 Spectrophotometer (Thermo Fisher Scientific, Waltham, MA, USA), and the extracted DNA was stored at −20 °C for further analysis.

### 4.3. LAMP Primer and Probe Design

The LAMP primer set comprised outer (F3 and B3) and inner (FIP and BIP) primers targeting six distinct regions of *vanA* in *Enterococcus* (GenBank accession no.: JN982328.1). For the LAMP-LFD assay, the DNA probe was labeled with fluorescein isothiocyanate (FITC) at the 5′ end, while the FIP was labeled with biotin. The primers and probe for detecting vancomycin-resistant *Enterococcus* were designed based on the vanA gene sequence using PrimerExplorer V5 (Eiken Chemical Co., Ltd., Tokyo, Japan). The probe was located between the B1 c and B2 regions to enable molecular hybridization for the detection of FITC–biotin-labeled LAMP amplicons. In silico analysis using BLASTN suggested high specificity of the designed primers and probe for the *vanA* gene. All primers and probes were synthesized by Pacific Science Co. Ltd. (Biobasic, Markham, ON, Canada).

### 4.4. PCR Assay Verification

In this study, two specific PCR primers, F3 and B3, were used to detect the *vanA* gene. PCR amplification was carried out in a 25 μL reaction mixture containing sterile water, 10× PCR buffer (Vivantis Technologies Sdn. Bhd., Selangor, Malaysia), 0.4 μM of F3 and B3 primers, 2.8 mM of MgCl_2_ (Vivantis Technologies Sdn. Bhd., Selangor, Malaysia), 0.2 mM of dNTPs (New England Biolabs, Ipswich, MA, USA), 2 units of Taq DNA polymerase (Vivantis Technologies Sdn. Bhd., Selangor, Malaysia), and 1 μL of DNA template. Milli-Q water was served as a blank negative control. The DNA samples, as well as a positive control (*E. faecium* ATCC 700221), were amplified *vanA* with an initial denaturation step of 5 min at 95 °C, followed by 35 cycles of 92 °C for 30 s where annealing temperature varied from 50 to 59 °C (1 °C interval) for 30 s and 72 °C for 1 min, and a final extension step at 72 °C for 5 min in a BIO-RAD T100 Thermal Cycler (Bio-Rad Laboratories, Inc., Hercules, CA USA). PCR products were analyzed via 2.0% agarose gel electrophoresis (AGE) at 100 volts for 30 min, stained with NEOgreen Gel Stain (NeoScience Co., Ltd., Suwon, Republic of Korea), and observed under a GelDoc Go Imaging System (Bio-Rad Laboratories, Hercules, CA USA).

### 4.5. LAMP Reaction Conditions

The LAMP reaction was conducted in a 25 μL reaction mixture containing sterile water, 1× supplied buffer (New England Biolabs, Ipswich, MA, USA), 2.0 μM each of FIP and BIP, 0.2 μM each of F3 and B3, 1.6 mM dNTPs (New England Biolabs, Ipswich, MA, USA), 0.5 M betaine (Sigma-Aldrich, St. Louis, MO, USA), 8 units of Bst 2.0 DNA polymerase (New England Biolabs, Ipswich, MA, USA), and a DNA template. Different reaction conditions were evaluated to optimize the LAMP reaction, including temperature (61 °C, 62 °C, 63 °C, 64 °C, 65 °C), reaction duration (15, 20, 25, 30, 45, 60 min), and MgSO_4_ concentration (4.0, 4.5, 5.0, 5.5 mM); in all experiments, the template DNA concentration was maintained at 50 ng. The LAMP assay was incubated in either a Thermal Cycler or a Dry Bath incubator (Hangzhou Allsheng Instruments Co., Ltd., Hangzhou, China), and Milli-Q water was used as a negative control to check for cross-contamination. LAMP products were analyzed via 2.0% AGE at 100 volts for 30 min, stained with NEOgreen Gel Stain (NeoScience Co., Ltd., Suwon, Republic of Korea). The LAMP products were then observed under a GelDoc Go Imaging System.

### 4.6. LAMP–Lateral Flow Dipstick (LAMP-LFD)

The primer set used for LAMP-LFD was identical to that of the standard LAMP assay, except that the forward inner primer (FIP) was labeled with biotin at the 5′ end. For detection, LAMP amplicons were hybridized with an FITC-labeled DNA probe. Briefly, 10 µL of LAMP product was mixed with 1 µL of FITC-DNA probe (5, 10, or 20 pmol) and incubated at 60 °C for 5, 10, or 15 min. Subsequently, 10 µL of the hybridized product was diluted in 100 µL of running buffer, and the LFD strip was inserted into the mixture for 5 min.

**Figure 6:**
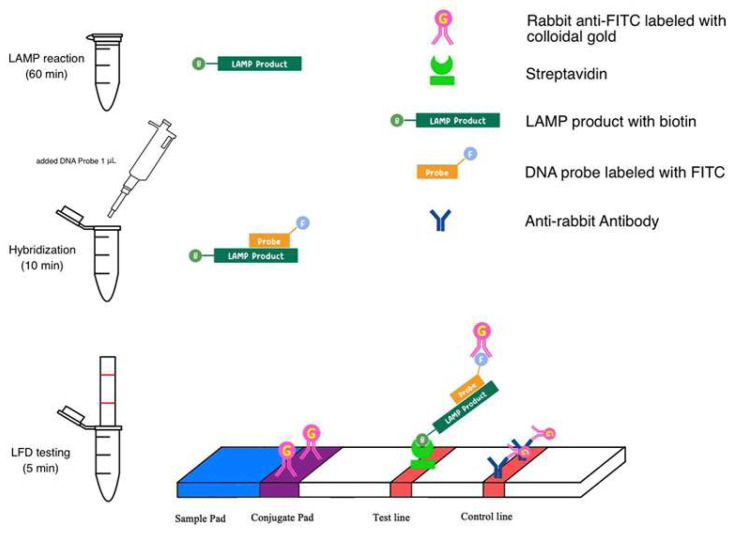
Detection of LAMP Products Using a LAMP–Lateral Flow Dipstick (LAMP-LFD). 10 microliters of LAMP product was mixed with 1 µL of FITC-DNA probe and incubated at 60 °C for 5 min. The mixture was then applied to the lateral flow dipstick. Following migration by capillary action, the appearance of both the test line and control line indicated a positive result, whereas the appearance of only the control line indicated a negative result.

During migration by capillary action, the biotin-labeled LAMP amplicons hybridized with the FITC probe and formed complexes with gold-labeled anti-FITC antibodies. A red-purple control line was observed in all strips, confirming proper assay performance. Assay results were visually interpreted within 5 min of sample application. A positive result was defined by the simultaneous appearance of two distinct red bands at both the Test Line (T-line) and the Control Line (C-line), indicating successful target amplification and capture. A negative result was characterized by a single visible band at the C-line with no color development at the T-line. The assay was considered invalid if no band appeared at the C-line, requiring a procedural repeat (Figure 6).

### 4.7. Analytical Specificity of PCR, LAMP, and LAMP-LFD

The analytical specificity of the PCR, LAMP, and LAMP–LFD assays was evaluated using genomic DNA extracted from 12 non-target bacterial strains commonly isolated from clinical urine specimens, including five Gram-positive bacteria—*Staphylococcus aureus* ATCC^®^ 25923, oxacillin-resistant *S. aureus* ATCC^®^ 43300 (MRSA), *E. faecalis* ATCC^®^ 29212, vancomycin-resistant *E. faecalis* ATCC^®^ 51299 (harboring *vanB* gene), and *Streptococcus pneumoniae* ATCC^®^ 49619—and seven Gram-negative bacteria—*Escherichia coli* ATCC^®^ 25922, *Acinetobacter lwoffii* ATCC^®^ 15309, *Enterobacter cloacae* ATCC^®^ 23355, carbapenem-resistant *Klebsiella pneumoniae* ATCC^®^ BAA-1705 (CRE), *K. aerogenes* ATCC^®^ 13048, *Pseudomonas aeruginosa* ATCC^®^ 27853, and *Burkholderia cepacia* ATCC^®^ 25416. Genomic DNA from *Enterococcus faecium* ATCC 700221 carrying the *vanA* gene was used as the positive control, and nuclease-free water served as the no-template control.

Each DNA sample was tested under the optimized PCR, LAMP, and LAMP–LFD conditions described above. PCR amplification products were analyzed by agarose gel electrophoresis, whereas LAMP products were detected by agarose gel electrophoresis or lateral flow dipstick, respectively. The specificity of each assay was determined based on the presence or absence of the expected amplification product or positive LFD signal.

All specificity experiments were performed in triplicate in separate runs. The results obtained for the non-target strains were compared with those of the *vanA*-positive control and the no-template control.

### 4.8. Analytical Sensitivity

The analytical sensitivity (limit of detection) of the PCR, LAMP and LAMP-LFD assays was measured using ten-fold serial dilutions of the positive control, genomic DNA of vancomycin-resistant *E. faecium* ATCC^®^ 700221, from 285.18 ng/µL to 2.852 fg/µL. This dilution was then tested using PCR, LAMP and LAMP-LFD assays. The LOD was defined as the lowest DNA concentration that consistently produced the expected amplification product by PCR or a positive result by LAMP and LAMP–LFD. All sensitivity experiments were performed in triplicate in separate runs. The results obtained for the non-target strains were compared with those of the *vanA*-positive control and the no-template control.

### 4.9. Application of LAMP-LFD Assay in Clinical Specimens

To evaluate the clinical performance of the LAMP–LFD assay, genomic DNA extracted from 52 *vanA*-positive vancomycin-resistant *Enterococcus* (VRE) isolates and 48 non-VRE clinical isolates was analyzed using PCR, LAMP, and LAMP–LFD under the optimized assay conditions described above. PCR was used as the reference method for comparison. The results obtained by LAMP and LAMP–LFD were compared with those of PCR to determine the concordance between the assays. Diagnostic performance was evaluated by calculating sensitivity, specificity, positive predictive value (PPV), negative predictive value (NPV), and overall accuracy using a 2 × 2 contingency table according to the *Methods Guide for Medical Test Reviews* [24].

## 5. Conclusions

In conclusion, a loop-mediated isothermal amplification coupled with a lateral flow dipstick (LAMP–LFD) assay was successfully developed for the rapid molecular detection of the *vanA* gene in vancomycin-resistant *Enterococcus*. The assay demonstrated complete agreement with the reference PCR method in the clinical isolates evaluated and provided results within approximately 1–1.5 h, including DNA extraction. In addition, the assay enables visual interpretation of amplification products without the need for gel electrophoresis or sophisticated laboratory equipment, making it a simple and potentially cost-effective molecular screening method for laboratories with limited diagnostic infrastructure. Nevertheless, the findings should be interpreted in light of the study limitations, including the relatively small sample size and single-center study design. Furthermore, although PCR was used as the molecular reference method, additional validation against phenotypic antimicrobial susceptibility testing using larger and more diverse clinical isolate collections is required before routine clinical implementation.

Future studies should investigate the feasibility of adapting the LAMP–LFD assay for point-of-care testing and evaluate its performance in multicenter clinical settings. The potential application of this platform for detecting other clinically important antimicrobial resistance genes also warrants further investigation.

## Figures and Tables

**Table 1:** The results of PCR, LAMP, and LAMP-LFD for the detection of *vanA* in clinical isolates of *Enterococcus* spp. (see Supplementary Figures S1–S3).

	PCR Positive	PCR Negative	Total
LAMP & LAMP-LFD positive	52	0	52
LAMP & LAMP-LFD negative	0	48	48
Total isolates	52	48	100

**Table 2:** Diagnostic performance of LAMP-LFD, with 95% confidence intervals, for *vanA* detection in 100 clinically isolated enterococci.

Parameter	Estimate (%)	95% CI (%)	Likelihood Ratios
Sensitivity	100	93.2–100	-
Specificity	100	92.6–100	-
PPV (Positive Predictive Value)	100	93.2–100	-
NPV (Negative Predictive Value)	100	92.6–100	-
Accuracy	100	96.4–100	-
LR+	-	-	∞
LR−	-	-	0

LR+ was mathematically infinite (∞) because no false-positive results were observed in the study population. This estimate should be interpreted cautiously because it is based on a relatively limited sample size and was calculated using PCR as the molecular reference method.

## Data Availability

The data presented in this study are available within the article and its Supplementary Materials.

## Supplementary Materials

The following supporting information can be downloaded at: https://www.mdpi.com/article/10.3390/ijms27156575/s1.

## Abbreviations

The following abbreviations are used in this manuscript:

°C	Degree(s) Celsius
AGE	agarose gel electrophoresis
ATCC	American Type Culture Collection
CRE	carbapenem-resistant *Enterobacterales*
DNA	deoxyribonucleic acid
fg/μL	femtograms per microliter
LAMP	loop-mediated isothermal amplification
LFD	lateral flow dipstick
MgCl_2_	magnesium chloride
MgSO_4_	magnesium sulfate
MRSA	methicillin-resistant *Staphylococcus aureus*
NPV	negative predictive value
PCR	polymerase chain reaction
pg/μL	picograms per microliter
PPV	positive predictive value
UV	ultraviolet
*vanA*	*vanA*-type of vancomycin-resistant enterococci
*vanB*	*vanB*-type of vancomycin-resistant enterococci
VRE	vancomycin-resistant enterococci
VSE	vancomycin-susceptible enterococci
WHO	World Health Organization
