# Supplementary material for: Development of an Assay Involving Loop-Mediated Isothermal Amplification with a Lateral Flow Dipstick for Detection of the vanA Gene in Vancomycin-Resistant Enterococci
Source: Int J Mol Sci. 2026 Jul 23;27(15):6575. doi: 10.3390/ijms27156575 (PMC13464955; doi:10.3390/ijms27156575)
Supplement: Supplementary file 1 [file ijms-27-06575-s001.zip › ijms-4383866-supplementary.pdf]

Figure S1. Detection of the *vanA* Gene in 100 Clinically Isolated Enterococci Using the PCR Assay. Representative PCR results obtained by 2% agarose gel electrophoresis from 100 clinical enterococcal isolates. Of the 100 isolates tested, 52 were positive for the *vanA* gene, exhibiting the expected 204-bp amplification product, whereas 48 were negative, showing no specific amplification. The PCR assay served as the reference method for evaluating the diagnostic performance of the LAMP-LFD assay.

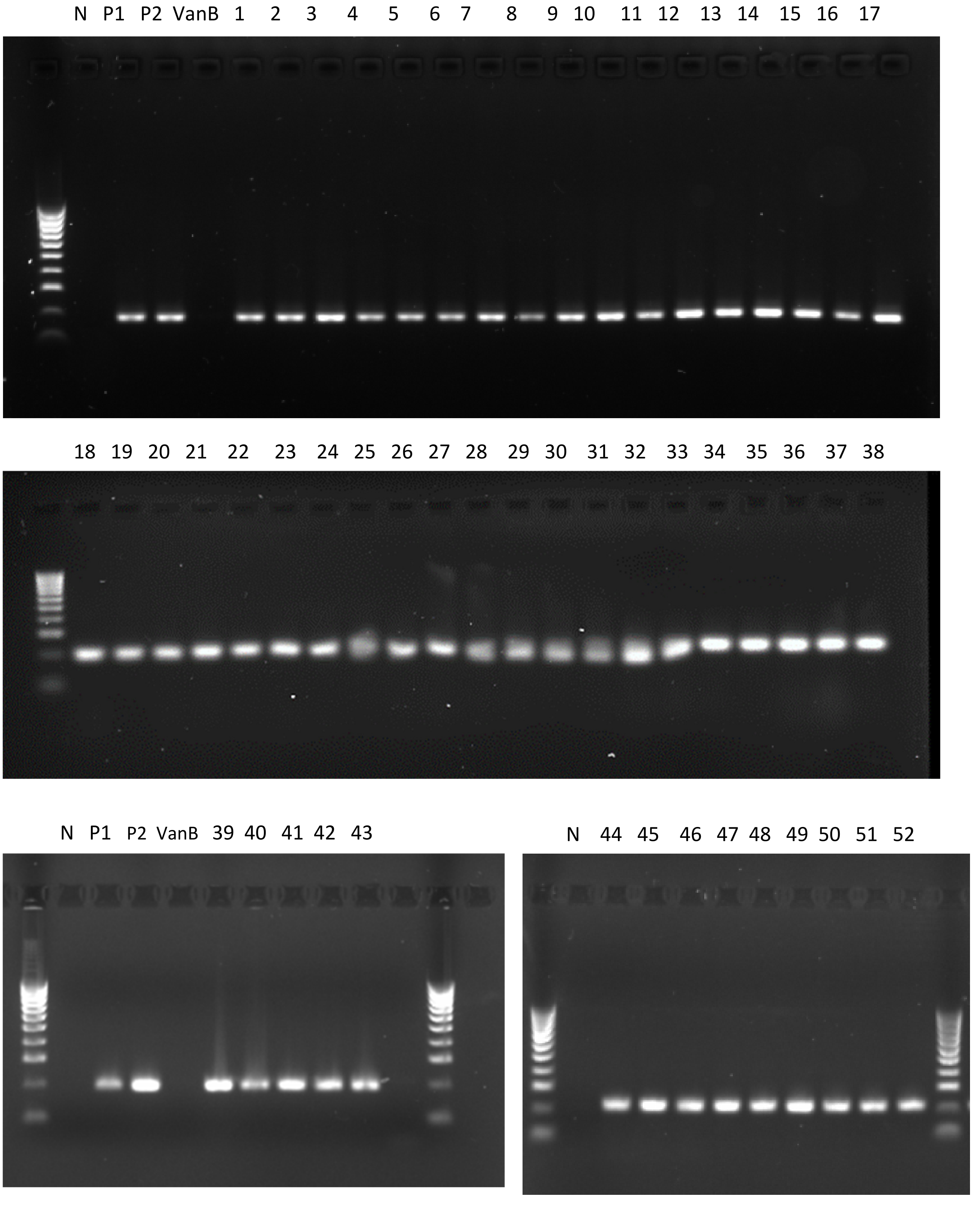

P1 N 53 54 55 54 55 56 57 58 59 60 61 62 63 64 65 66 67 68 69

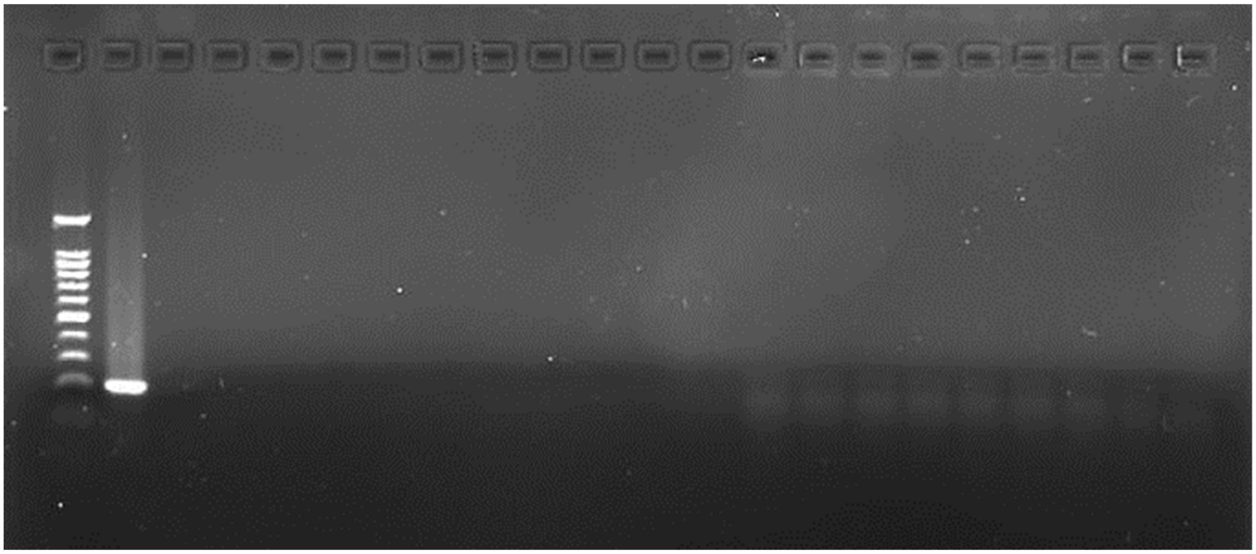

P N 70 71 72 73 74 75 76 77 78 79 80 81 82 83 84 85 86 87 88

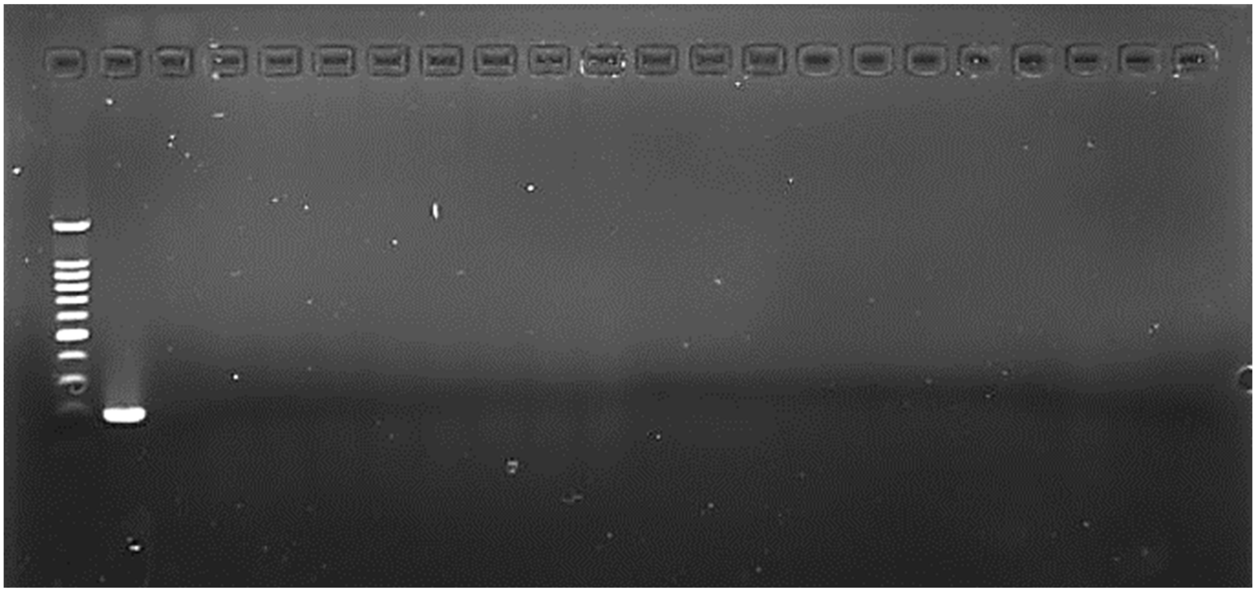

Figure S2. Detection of the *vanA* Gene in 100 Clinically Isolated Enterococci Using the LAMP Assay. Representative LAMP results obtained by 2% agarose gel electrophoresis from 100 clinical enterococcal isolates. Of the 100 isolates tested, 52 were positive for the *vanA* gene, exhibiting the characteristic ladder-like banding pattern of LAMP amplification, whereas 48 were negative, showing no amplification products. The LAMP results were in complete agreement with the reference PCR assay.

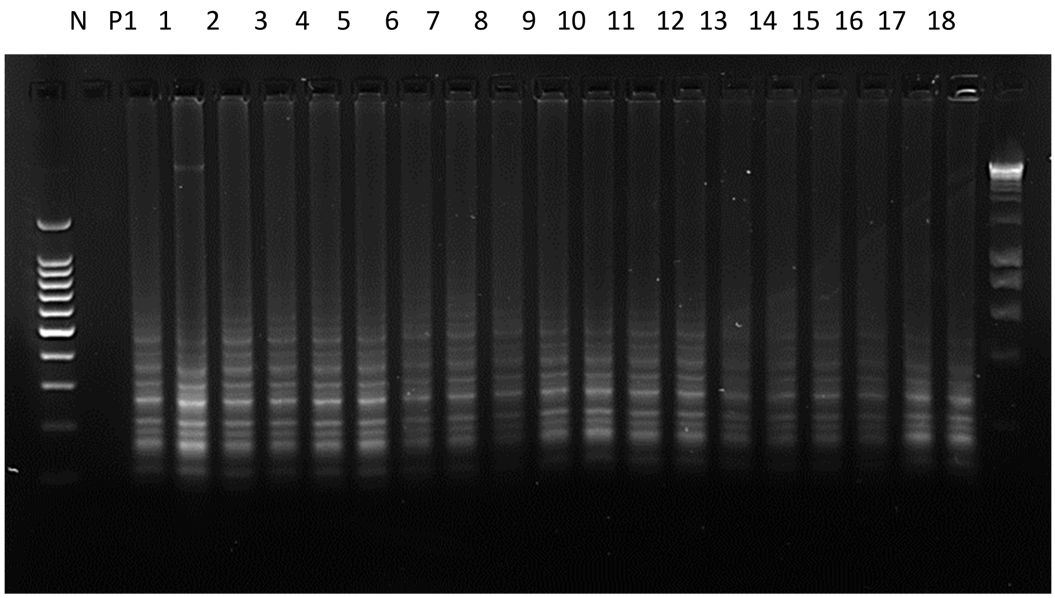

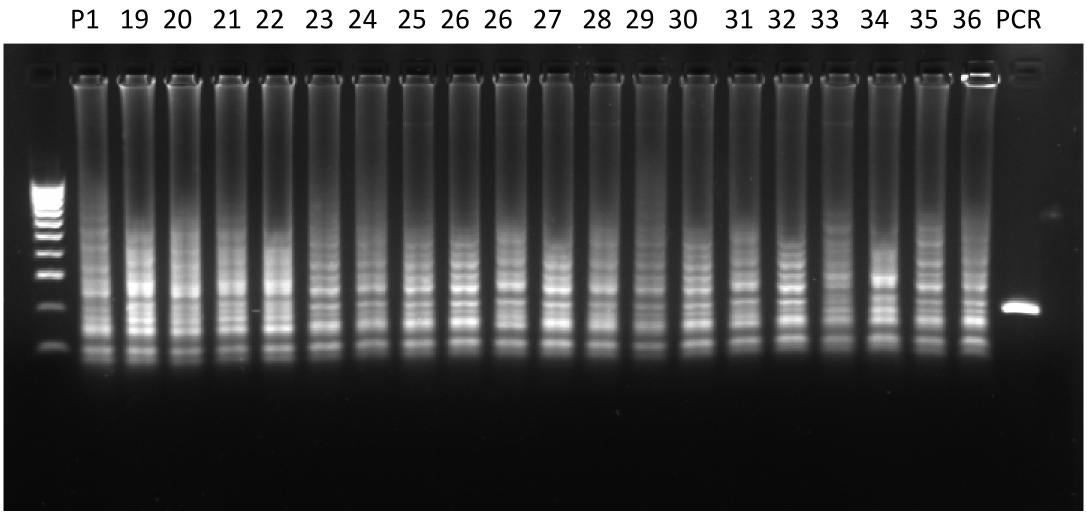

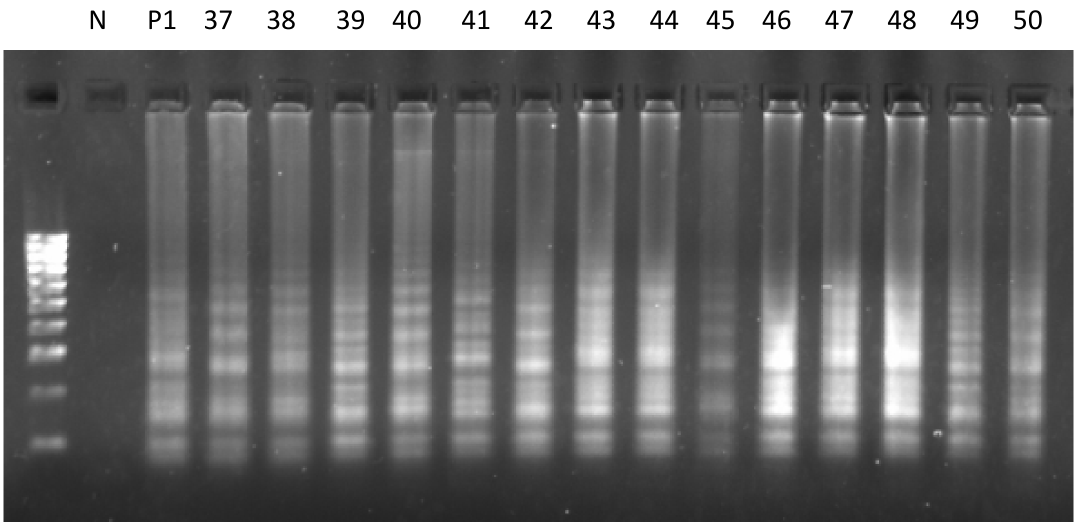

P1 P2 51 52 N 53 54 55 56 57

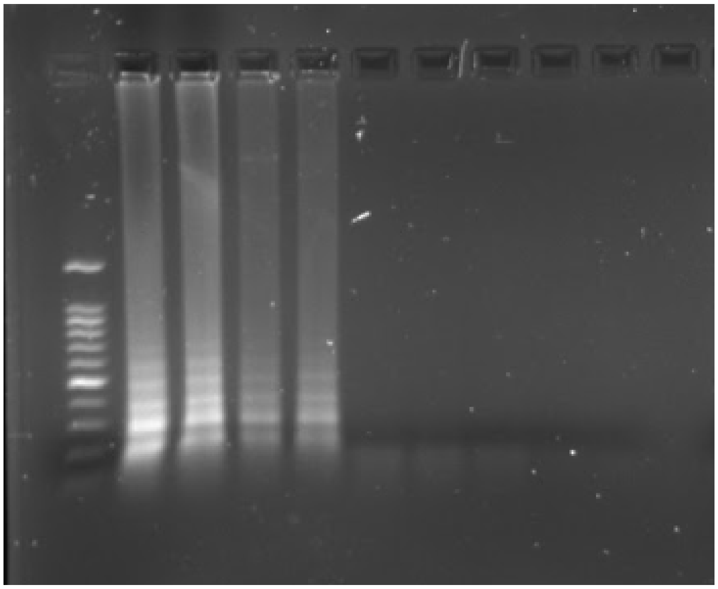

N P1 58 59 60 61 62

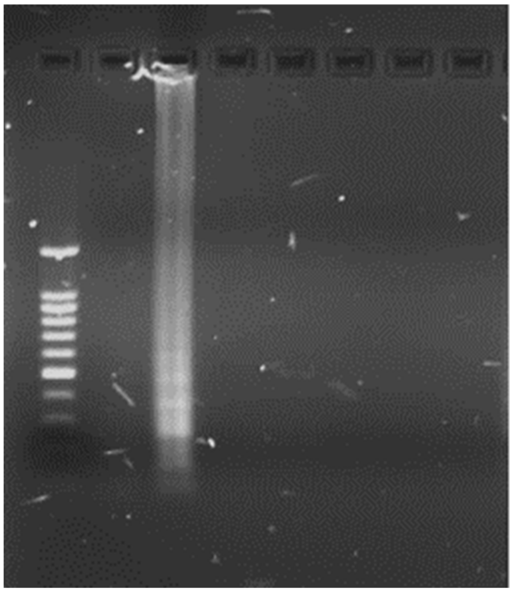

P1 N 63 64 65 66 67 68 69 70 71 72 73 74 75 76 77 78 79 80 81

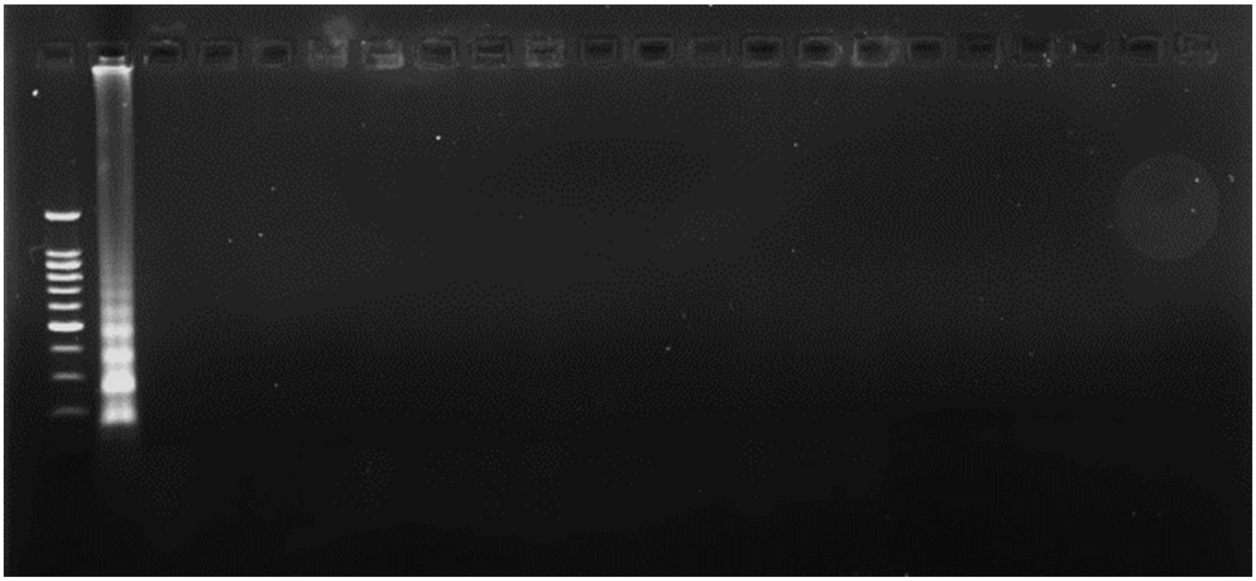

P1 N 82 83 84 85 86

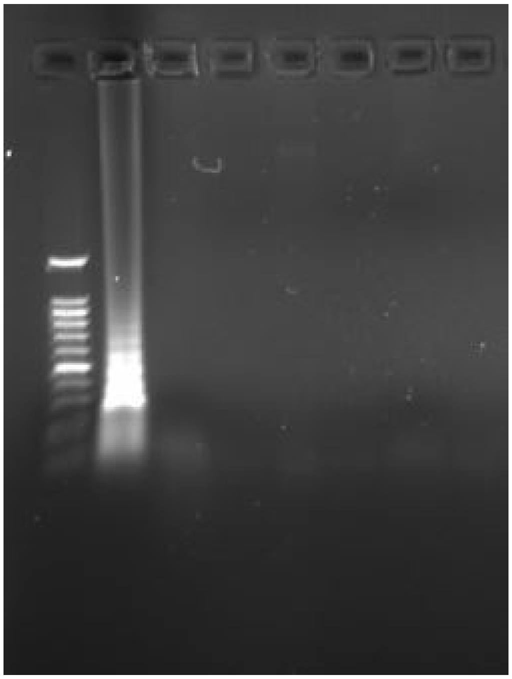

N P1 87 88 89 90 91 92 93 94 95

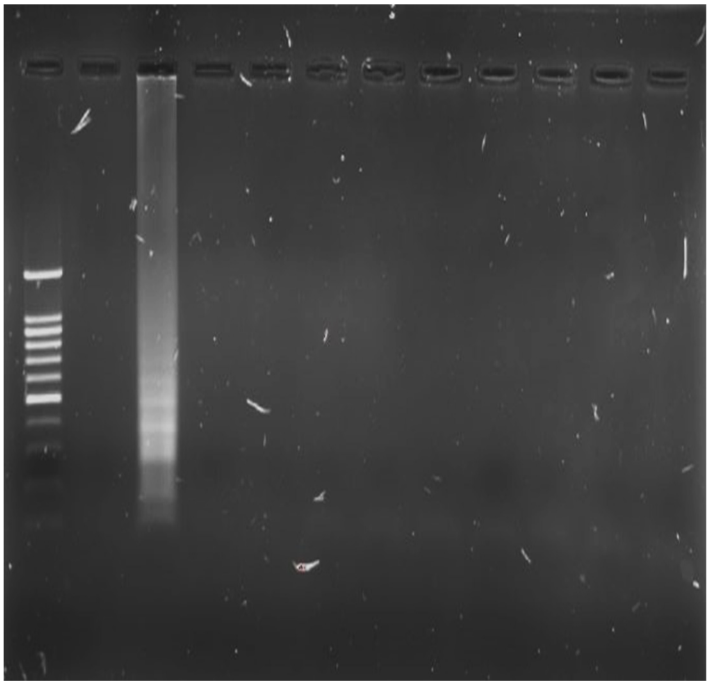

P1 N 82 83 84 85 86 87 88 89 90 91 92 93 94 95 96 97 98 99 100

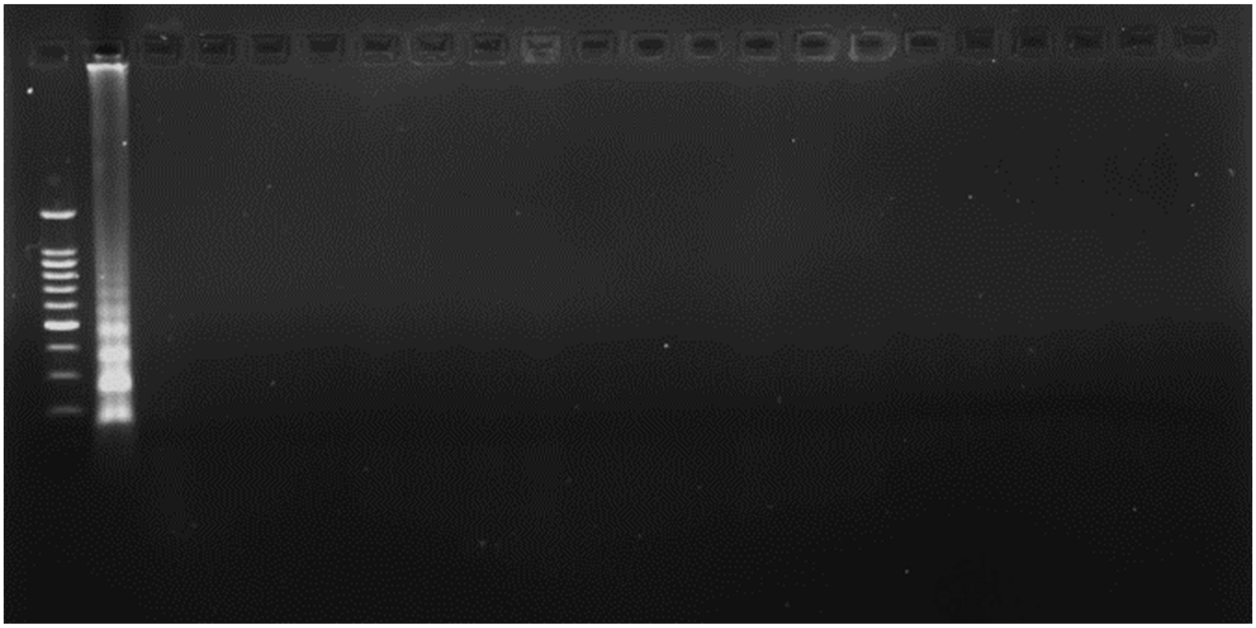

Figure S3. Detection of the *vanA* Gene in 100 Clinically Isolated Enterococci Using the LAMP–Lateral Flow Dipstick (LAMP-LFD) Assay. Representative LAMP-LFD results obtained from 100 clinical enterococcal isolates. Of the 100 isolates tested, 52 were positive for the *vanA* gene, showing both the test line and the control line, whereas 48 were negative, showing only the control line. The LAMP-LFD results were in complete agreement with the reference PCR assay, demonstrating 100% sensitivity and 100% specificity for *vanA* detection.

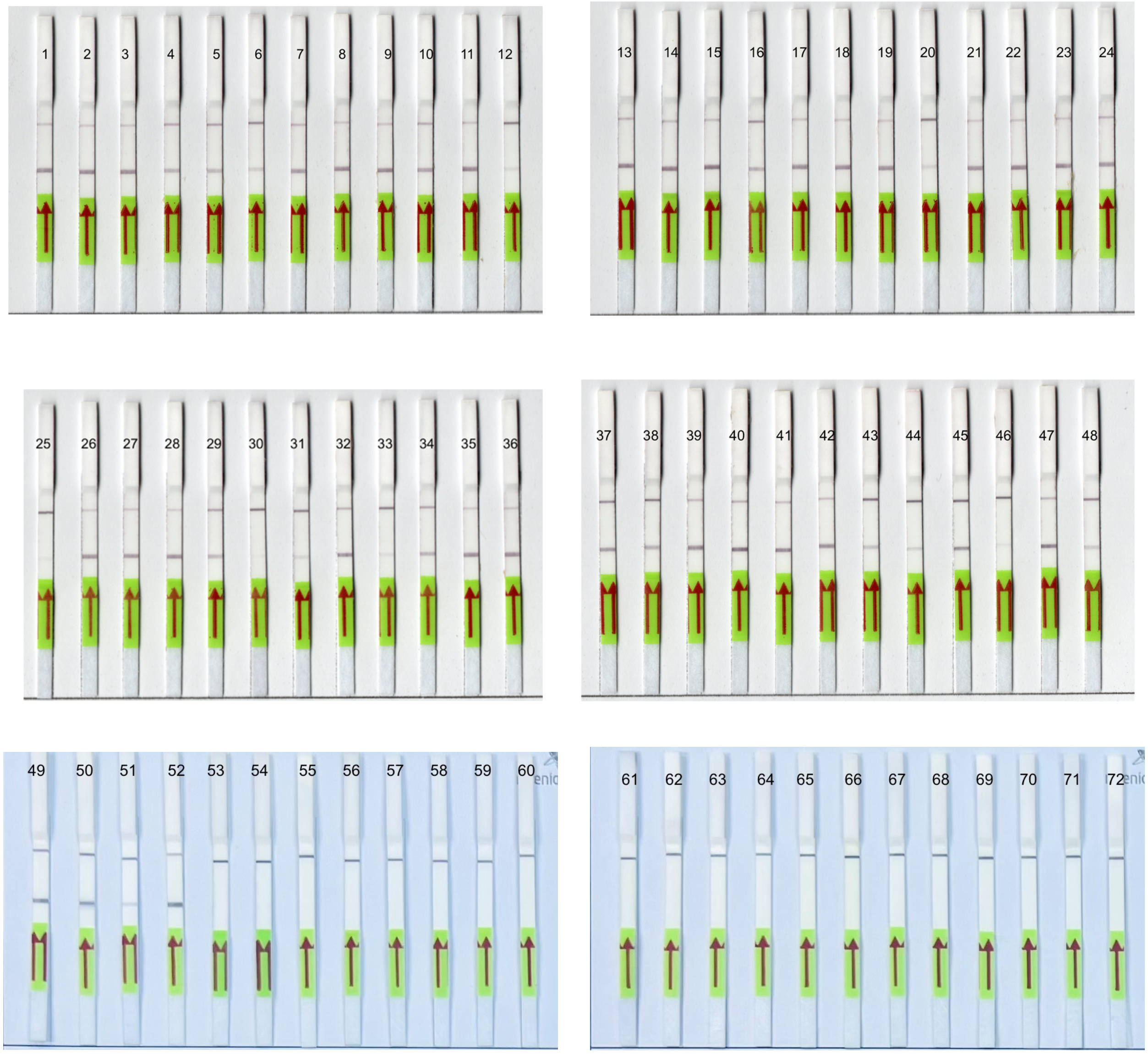

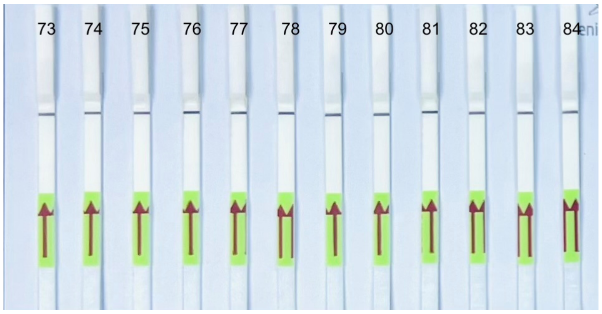
73 74 75 76 77 78 79 80 81 82 83 84

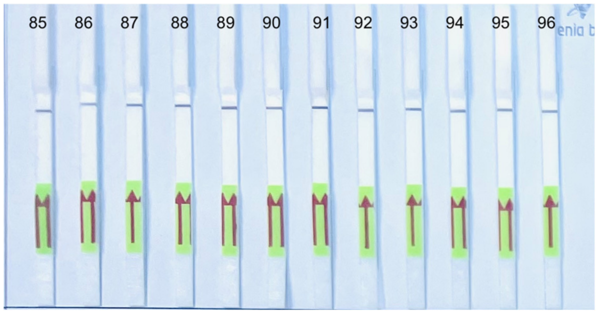
85 86 87 88 89 90 91 92 93 94 95 96

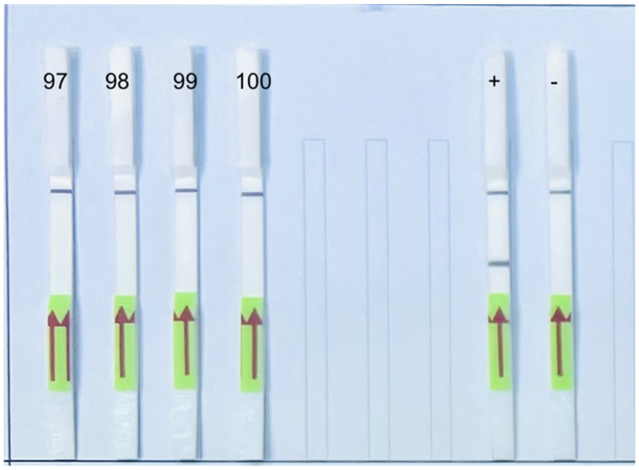
97 98 99 100
+ -
